# Yogurt consumption and risk of mortality from all causes, CVD and cancer: a comprehensive systematic review and dose–response meta-analysis of cohort studies

**DOI:** 10.1017/S1368980022002385

**Published:** 2023-06

**Authors:** Helda Tutunchi, Sina Naghshi, Mohammad Naemi, Fatemeh Naeini, Ahmad Esmaillzadeh

**Affiliations:** 1 Endocrine Research Center, Tabriz University of Medical Sciences, Tabriz, Iran; 2 Students’ Scientific Research Center, Tehran University of Medical Sciences, Tehran, Iran; 3 Department of Clinical Nutrition, School of Nutritional Sciences and Dietetics, Tehran University of Medical Sciences, Tehran, Iran; 4 Department of Community Nutrition, School of Nutritional Sciences and Dietetics, Tehran University of Medical Sciences, 14155-6117 Tehran, Iran; 5 Obesity and Eating Habits Research Center, Endocrinology and Metabolism Molecular – Cellular Sciences Institute, Tehran University of Medical Sciences, Tehran, Iran; 6 Department of Community Nutrition, School of Nutrition and Food Science, Isfahan University of Medical Sciences, Isfahan, Iran

**Keywords:** Dairy, Yogurt, Mortality, Death, Cancer, Meta-analysis, Cohort study

## Abstract

**Objectives:**

To quantify the dose–response relation between yogurt consumption and risk of mortality from all causes, CVD and cancer.

**Design:**

Systematic review and meta-analysis.

**Setting:**

We conducted a comprehensive search of PubMed/Medline, ISI Web of Science and Scopus databases through August 2022 for cohort studies reporting the association of yogurt consumption with mortality from all causes, CVD and cancer. Summary relative risks (RR) and 95 % CI were calculated with a random-effects model.

**Participants:**

Seventeen cohort studies (eighteen publications) of 896 871 participants with 75 791 deaths (14 623 from CVD and 20 554 from cancer).

**Results:**

High intake of yogurt compared with low intake was significantly associated with a lower risk of deaths from all causes (pooled RR 0·93; 95 % CI: 0·89, 0·98, I^2^ = 47·3 %, *n* 12 studies) and CVD (0·89; 95 % CI: 0·81, 0·98, I^2^ = 33·2 %, *n* 11), but not with cancer (0·96; 95 % CI: 0·89, 1·03, I^2^ = 26·5 %, *n* 12). Each additional serving of yogurt consumption per d was significantly associated with a reduced risk of all-cause (0·93; 95 % CI: 0·86, 0·99, I^2^ = 63·3 %, *n* 11) and CVD mortality (0·86; 95 % CI: 0·77, 0·99, I^2^ = 36·6 %, *n* 10). There was evidence of non-linearity between yogurt consumption and risk of all-cause and CVD mortality, and there was no further reduction in risk above 0·5 serving/d.

**Conclusion:**

Summarising earlier cohort studies, we found an inverse association between yogurt consumption and risk of all-cause and CVD mortality; however, there was no significant association between yogurt consumption and risk of cancer mortality.

Annually, more than half of the deaths are due to CVD and cancer worldwide^(1,2)^. Healthy diet plays a prominent and profound role in these conditions. Yogurt, a dairy product, made from the fermentation of lactic acid in milk by *Lactobacillus bulgaricus* and *Streptococcus thermophilusis,* is among the most widely consumed fermented foods^(3)^. Due to the close contribution of gut microbiota to human health and the effect of yogurt consumption on gut microbiota, its consumption might affect health outcomes. Moreover, yogurt provides energy and beneficial compounds such as proteins, minerals, multivitamins and conjugated linoleic acid that may jointly favour long-term health^(4)^. However, it is also a source of saturated fats and added sugar, which is presumed to increase the risk of CVD and mortality^(2,5)^.

Several epidemiological studies have previously examined the association between yogurt consumption and risk of chronic diseases^(6)^. Regular consumption of yogurt has been associated with a lower risk of hypertension^(7)^, type 2 diabetes^(8)^ and certain cancers^(9,10)^. However, findings on the contribution of yogurt consumption to longevity are limited and conflicting^(11)^. While some prospective studies have indicated an inverse association between yogurt intake and overall mortality^(12,13)^, some others have failed to reach such findings^(14,15)^. Moreover, different associations were reported between yogurt consumption and mortality outcomes in men and women^(13,16)^. In an earlier meta-analysis of prospective cohort studies, high yogurt intake was not related to a lower risk of all-cause, CVD and cancer mortality^(17)^. However, this review had several limitations. For example, it missed several large and some smaller studies^(14,16,18–22)^. In addition, two ineligible studies were included in the analysis^(23,24)^. The exposure in one of these studies was a combination of dairy products not just yogurt^(23)^, and in the other study, patients with cancer were included^(24)^. Furthermore, several studies have since been published^(13,25–28)^. Given the above-mentioned points, a comprehensive review and meta-analysis is required to quantitatively summarise earlier studies in this regard. The present study was therefore performed to comprehensively examine the association between the contribution of yogurt consumption to the risk of mortality from all causes, CVD and cancer based on earlier publications and to do a dose–response meta-analysis of cohort studies in this regard.

## Methods

The present study was performed based on Preferred Reporting Items for Systematic Reviews and Meta-Analyses (PRISMA) guidelines^(29)^.

### Search strategy

All relevant studies were identified by searching PubMed/Medline, ISI Web of Science and Scopus databases, without any language restriction, from inception to August 2022. Two independent authors (SN and FN) developed and performed the literature search. The following keywords were used in our search strategy: ((‘dairy products‘(Mesh) OR ‘dairy products’(tiab) OR ‘dairy’(tiab) OR ‘yogurt’(Mesh) OR ‘yogurt’(tiab) OR ‘yoghurt” (tiab)) AND (‘mortality’(tiab) OR ‘death’(tiab) OR ‘fatal’(tiab) OR ‘survival’(tiab) OR ‘mortality’(Subheading) OR ‘neoplasm’(tiab) OR ‘cancer survivor’(tiab) OR ‘cardiovascular disease’(tiab) OR ‘coronary disease’(tiab) OR ‘myocardial ischemia’(tiab) OR ‘coronary artery disease’(tiab) OR ‘myocardial infarction’(tiab) OR ‘stroke’(tiab) OR ‘mortality’(Mesh) OR ‘death’(Mesh) OR ‘neoplasms’(Mesh) OR ‘cancer survivors’(Mesh) OR ‘cardiovascular diseases’(Mesh) OR ‘coronary disease’(Mesh) OR ‘myocardial ischemia’(Mesh) OR ‘coronary artery disease’(Mesh) OR ‘myocardial infarction’(Mesh) OR ‘stroke’(Mesh))). In addition, the bibliographies of the retrieved articles and previous systematic and narrative reviews were also scanned manually to identify potential publications.

### Inclusion criteria

Two independent authors (SN and FN) screened the title and abstract of publications found in the systematic search to identify eligible studies. The publications were considered for inclusion in this meta-analysis if they (1) were cohort studies (2) reported hazard ratios (HR) or relative risks (RR) and their corresponding 95 % CI for the association between yogurt consumption and mortality from all causes, CVD and cancer. If multiple papers were published using the dataset of a single cohort, we included the one with the most comprehensive information.

### Exclusion criteria

We excluded ecologic studies, non-original research papers (reviews, letters, editorials or commentaries) and meta-analyses. We also did not include unpublished studies as well as those studies where individuals were included based on the existence of a specific disease at baseline. In addition, if a study reported the risk estimates for yogurt consumption in combination with other dairy foods, it was not included in our analysis.

### Data extraction

Using a standardised form, pairs of authors (HT and SN) independently reviewed the title and abstract of all articles and extracted the required data. In case of any disagreements, consensus was reached. For each study, we recorded the following information: the first author’s name, year of publication, study location, mean age or age range, sex, sample size, length of follow-up, type of outcome, number of deaths from all causes, CVD and cancer, dietary assessment method, the amount of yogurt consumption in each category, adjustment for confounding variables in multivariable analysis as well as RR and 95 % CI of mortality for each category of yogurt consumption. We extracted RR with the most adjusted model. For one study that reported multivariable models with and without additional adjustment for dietary Ca intake, we selected the multivariable model without adjustment for dietary Ca intake.

### Risk of bias assessment

Quality assessments were performed in duplicate by two independent reviewers (SN and FN). The Newcastle–Ottawa scale was used to assess the quality of each study^(30)^. The total score of Newcastle–Ottawa scale ranges between 0 and 9. Studies achieving nine points were considered to provide the highest quality.

### Data synthesis and analysis

Reported RR and HR and their 95 % CI for comparison of the highest *v*. the lowest categories of yogurt consumption were applied to estimate log RR and HR ± se. If a study reported risk estimates per standard deviation (sd) or per unit increment in yogurt consumption, the following method was used to translate per sd or per unit increment risk estimate to the top compared with the bottom categories of population baseline distribution of yogurt values. Briefly, we calculated the difference between the means/medians of the highest and lowest categories of yogurt consumption in other publications included in the analysis. Then, the mean difference between the mean/median of the highest and lowest categories was calculated. Finally, per-sd or per unit increase risk estimate was transformed to per ‘calculated mean/median difference’ and was included in the analysis. When the exact amount of sd was not presented in the published paper, we assumed a scaling factor of 2·18 times the log risk estimate for a 1-sd increase in yogurt consumption^(31)^. The analyses were conducted using a random-effects meta-analysis. Cochran’s Q test and I^2^ were used to estimate between-study heterogeneity. A value of *I* ^2^ more than 50 % was considered as substantial heterogeneity^(32)^. When a study provided results by sex, we first pooled these estimates using a fixed-effects model and included the pooled value in the main analysis, but sex-specific results were presented separately in subgroup analyses. For studies that reported results separately for CHD and stroke mortality or cancer subtypes, we used the method developed by Hamling et al^(33)^ to combine the risk estimates, and the obtained pooled risk estimate was included in the main meta-analysis. For studies that did not provide the information required to apply the Hamling method, we used a fixed-effects model to pool the results. Yogurt intake was converted into serving/d based on standard portion sizes developed by the US Department of Agriculture (i.e. one serving = 244 g)^(34)^.

In case of between-study heterogeneity, subgroup analysis was done based on study location, sex, follow-up duration, methods used for assessing dietary intakes and adjustment for confounding factors (BMI and energy intake) to find potential sources of heterogeneity. The possibility of publication bias was examined using the Egger’s and Begg’s tests as well as visual inspection of funnel plots^(35)^. The sensitivity analysis was conducted to find the effect of any specific study on the pooled effect size.

A method suggested by Greenland and Longnecker^(36)^ and Orsini et al^(37)^ was used to examine dose–response analysis. We computed study-specific slopes (linear trends) and 95 % CI from the natural logarithm of the RR, or HR, and their corresponding CI across categories of yogurt consumption. In this method, information on the number of deaths, number of participants and the effect sizes with the variance estimates for ≥ 3 quantitative categories of exposure was required. We used the median or mean amount of each category. For studies that reported the range of yogurt consumption rather than mean, the midpoint of the upper and lower limits was used to determine the amount of yogurt consumption. If the highest and lowest categories of yogurt consumption were open-ended, the width of the adjacent interval was used to calculate an upper or lower cut-off value.

A two-stage generalised least-squares trend estimation method was applied to assess a linear dose–response association of each additional serving of yogurt consumption with mortality risk. In this method, the study-specific slope lines are estimated and combined with a random-effects model to obtain an overall average slope^(37)^. To assess a non-linear dose–response association, restricted cubic splines with three knots at fixed percentiles of 10, 50 and 90 % of the distribution were applied^(36)^. First, a restricted cubic spline model with a generalised least-squares trend estimation method was calculated after taking into account the correlation within each set of reported RR/HR. Second, all the study-specific estimates were combined with the use of the restricted maximum likelihood method in a multivariate random-effects meta-analysis^(37,38)^. A probability value for non-linearity was evaluated by null hypothesis testing in which the coefficient of the second spline was considered equal to zero. All statistical tests were performed using the Stata software version 14 (Stata Corp. College Station, TX). For all tests, *P*-values less than 0·05 were considered statistically significant.

## Results

### Literature search

**Fig. 1:**
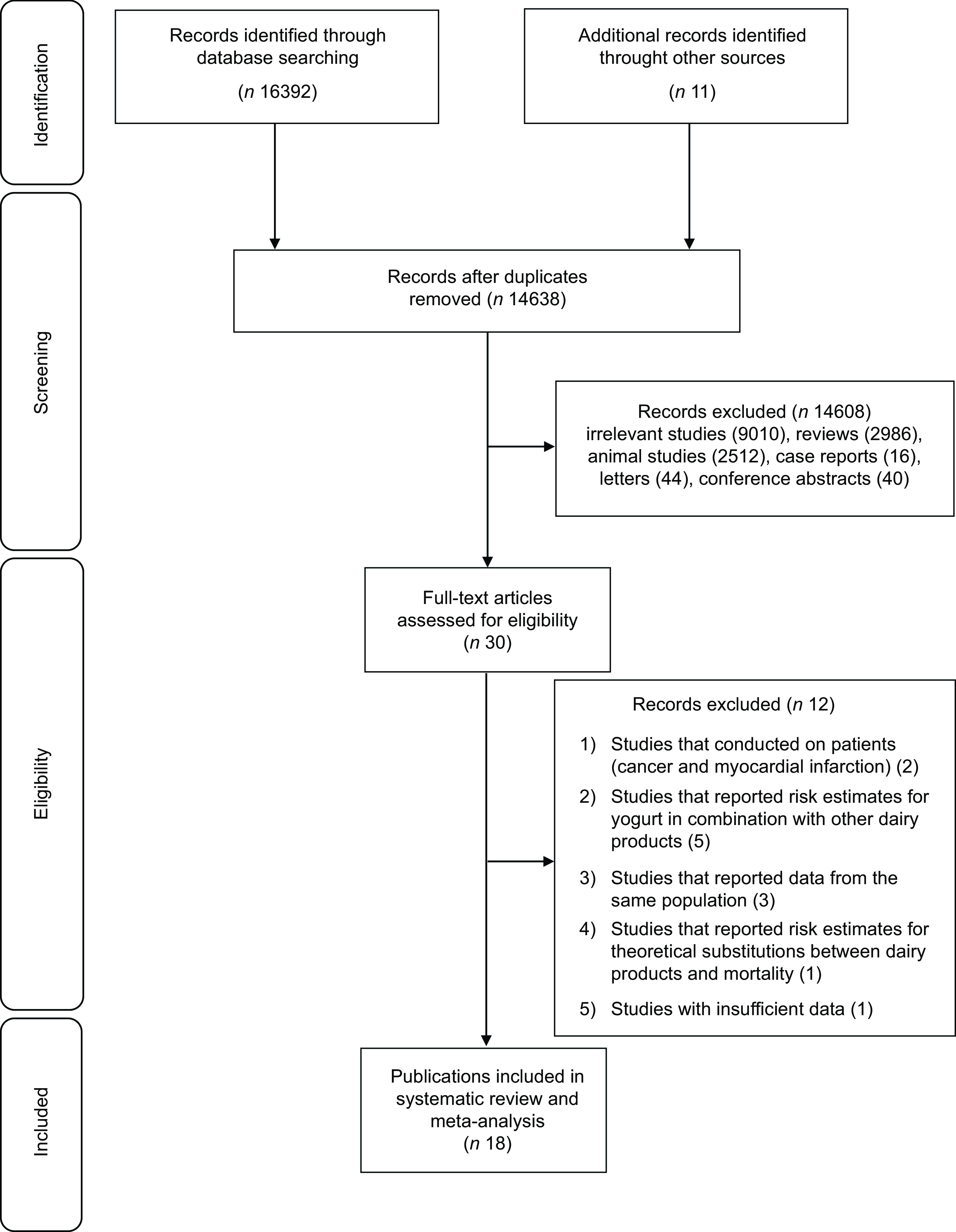
Flow diagram of study selection

As presented in Fig. 1, our search initially identified 16 392 publications, of which 14 638 were considered after removal of duplicate articles. Of these, 14 608 articles were again excluded, because they were irrelevant to the study objective. Finally, the full text of thirty papers were assessed, of them twelve publications were excluded: one reported risk estimates for theoretical substitutions between dairy products and mortality outcomes^(39)^, two was performed on colorectal cancer^(24)^ and myocardial infarction patients^(40)^, three were conducted on the same population with the same outcome^(5,15,41)^, five studies had reported the risk estimates for combination of dairy products, not yogurt separately^(23,42–45)^, and one study had insufficient data^(46)^. Finally, we considered data from seventeen cohort studies (eighteen publications)^(2,12–14,16,18–22,25–28,47–50)^. One cohort study in Japan had three publications on colorectal^(19)^, stomach^(21)^ and ovarian^(22)^ cancer mortality. The article of Schmid et al^(13)^ was published on two cohort studies including Nurses’ Health Study (NHS) and Health Professionals Follow-up Study (HPFS). Out of these, eleven publications had considered all-cause mortality, ten were about CVD mortality and thirteen about cancer mortality.

### Characteristics of included studies

Table 1 provided detailed information for each eligible study. Among seventeen cohort studies published between 2004 and 2022 with a follow-up period of 6–32 years, four (three publications) were conducted in the USA^(13,18,27)^, one in the United Kingdom^(50)^, three in the Netherlands^(14,16,49)^, one in Australia^(47)^, five (seven publications and three of them were reports from one study) in Japan^(19–22,26,28,48)^, one in Italy^(25)^, one in Iran^(12)^ and the study of Dehghan et al^(2)^ on population from twenty-one different countries. The sample size of the included studies varied from 1529 to 293 888 participants with an age range of 18–90 years. Overall, 896 871 individuals with 75 791 cases of all-cause mortality, 20 554 cases of cancer mortality and 14 623 cases of CVD mortality were included. One publication included only men^(18)^, one included only women^(22)^, and the others included both men and women. However, statistical analyses were separately done for males and females in six papers. In most studies, yogurt consumption was evaluated using FFQ, although one study had applied food recall^(27)^. All studies had adjusted for age. Moreover, most publications had controlled for BMI (*n* 14), physical activity (*n* 11), smoking status (*n* 14), alcohol intake (*n* 13), energy intake (*n* 11) and other dietary variables (*n* 10). Based on the Newcastle–Ottawa scale score^(30)^, thirteen papers which had a total score above the median (≥ 7) were defined to be high quality (online Supplementary Table 1).

**Table 1:** Characteristics of included studies on the association between yogurt consumption and mortality in adults aged > 18 years

Author	Cohort name	Country	Age	Sample size	Follow-up	Cases	Outcome	Exposure assessment	Comparison	RR	95 %CI	Adjustment
Soedamah-Muthu et al. 2013	Whitehall II	UK	35–55	M/F: 4522	11·7	237	All-cause mortality	FFQ	> 69 *v*. < 10 g/d	HR: 0·74	0·53, 1·05	Age, ethnicity and employment grade, smoking, alcohol intake, BMI, physical activity and family history of CHD/hypertension, fruit and vegetables, bread, meat, fish, coffee, tea and total energy intake
Bonthuis et al. 2010	NSCS	Australia	25–78	M/F: 1529	14·4	177	All-cause mortality	FFQ	> 30 *v*. < 2 g/d	HR: 1·20	0·79, 1·83	Age, sex, total energy intake, BMI, alcohol intake, school leaving age, physical activity level, pack years of smoking, dietary supplement use, b-carotene treatment during trial and presence of any medical condition.
						61	CVD mortality			HR: 0·71	0·31, 1·65	
Schmid et al. 2020	HPFS	USA	40–75	M: 40 278	26	12 397	All-cause mortality	FFQ	> 4 serv/week *v*. never	HR: 1·05	0·95, 1·16	Age, 2-year follow-up cycle, height, current BMI, BMI at the age of 18 years (women) or 21 years (men), ethnicity, physical activity, smoking status, pack-years of smoking, history of hypertension, history of hypercholesterolemia, history of diabetes, family history of cancer, family history of diabetes, family history of myocardial infarction, current multivitamin use, regular aspirin use, menopausal status and hormone use in women, total caloric intake, alcohol consumption, glycemic load, and intakes of unprocessed red meat, processed meat, nuts, total fibre, fruits, vegetables, and total Ca.
						3733	CVD mortality			HR: 0·95	0·79, 1·13	
						4000	Cancer mortality			HR: 1·10	0·93, 1·30	
	NHS		30–55	F: 82 348	32	20 831	All-cause mortality		> 4 serv/week *v*. never	HR: 0·91	0·85, 0·98	
						4207	CVD mortality			HR: 0·92	0·79, 1·08	
						7985	Cancer mortality			HR: 0·87	0·78, 0·98	
Farvid et al.2017	GCS	Iran	36–85	F/M: 42 403	11	3291	All-cause mortality	FFQ	≥ 0·75 *v*.≤ 0·2 serv/d	HR: 0·89	0·79, 1·00	Sex, age, ethnicity, education, marital status, residency, smoking, opium use, alcohol use, BMI, systolic blood pressure, occupational physical activity, family history of cancer, wealth score, medication use and energy intake.
						1467	CVD mortality			HR:0·84	0·70, 1·00	
						859	Cancer mortality			HR:0·86	0·69, 1·08	
Dehghan et al. 2018	PURE	21 countries	35–70	F/M: 123 830	9·1	6796	All-cause mortality	FFQ	> 1 serv *v*. zero	HR: 0·82	0·68, 0·98	Age, sex, education, urban or rural location, smoking status, physical activity, alcohol intake, history of diabetes, family history of CVD, family history of cancer, and quintiles of fruit, vegetable, red meat, starchy foods intake, and energy
Praagman et al. 2015	EPIC-NL	The Netherlands	20–70	F/M: 34 409	15	2436	All-cause mortality	FFQ	> 104 *v*. < 15 g/d	HR: 0·95	0·85, 1·07	Age, sex and total energy intake, smoking habit, BMI, physical activity, education level, hypertension at baseline, intakes of alcohol and energy-adjusted intakes of fruit and vegetables
						726	CVD mortality			HR:1·08	0·87, 1·34	
						1216	Cancer mortality			HR:1·02	0·86, 1·21	
Pala et al.2019	EPIC-Italy	Italy	50·1	F/M: 45 009	14·9	2468	All-cause mortality	FFQ	> 120 *v*. 0 g/d	HR:0·95	0·82, 1·09	Age, sex, centre, energy, weight, height, and waist-to-hip ratio; alcohol consumption; smoking status; physical activity; relative index of inequality; Italian Mediterranean Index; and intake of sugar.
						459	CVD mortality			HR:0·85	0·59, 1·23	
						1464	Cancer mortality			HR:1·00	0·83, 1·20	
Praagman et al.2014	RS	The Netherlands	> 55	F/M: 4235	17·3	576	Fatal CHD	FFQ	> 100 *v*. < 50 g/d	HR: 0·98	0·76, 1·26	Age, gender, and total energy intake, BMI, smoking, education level, intakes of alcohol, vegetables, fruit, meat, bread, fish coffee, and tea
						564	Fatal stroke			HR: 1·01	0·71, 1·44	
Park et al. 2007	NIH-AARP	USA	50–71	M:293 888	6	178	Prostate cancer mortality	FFQ	≥ 0·5 *v*. 0 serv/d	HR: 0·78	0·25, 2·50	Age; race/ethnicity; education; marital status; BMI; vigorous physical activity; smoking; alcohol consumption; history of diabetes; family history of prostate cancer; screening for prostate cancer by use of prostate-specific antigen; and intakes of tomatoes, red meat, fish, vitamin E, alpha-linolenic acid, and total energy.
Kojima et al. 2014	JACC	Japan	40–79	M: 45 181	9·9	79	Colon cancer mortality	FFQ	High *v*. low	HR: 0·80	0·42, 1·51	Age, family history of colorectal cancer, BMI, frequency of alcohol intake, current smoking status, walking time per d and educational level
						72	Rectal cancer mortality			HR: 0·46	0·21, 1·02	
			40–79	F: 62 643	9·9	97	Colon cancer mortality		High *v*. low	HR: 0·97	0·61, 1·56	
						26	Rectal cancer mortality			HR :1·51	0·60, 3·80	
Matsumoto et al. 2007	JMS	Japan	19–90	M/F: 11 606	9·15	255	Cancer mortality	FFQ	everyday *v*. not everyday	HR: 1·48	0·59, 3·72	Sex and age
Goldbohm et al. 2011	NLCS	The Netherlands	55–69	M: 12 912	10	10 658	All-cause mortality	FFQ	per 100 ml/d	RR: 0·96	0·92, 1·00	Age, education, cigarette smoking; nonoccupational physical activity, BMI, multivitamin use, alcohol, energy, energy-adjusted mono- and polyunsaturated fat intakes, and vegetable and fruit consumption.
						520	Stroke mortality			RR: 0·68	0·51, 0·91	
			55–69	F: 7870	10	5478	All-cause mortality		per 100 ml/d	RR: 1·00	0·95, 1·05	
						322	Stroke mortality			RR: 0·70	0·54, 0·92	
Sakauchi et al. 2007	JACC	Japan	40–79	F: 64 327	13·3	47	Ovarian cancer mortality	FFQ	> 1–2 times/week *v*. seldom	HR: 1·66	0·71, 3·91	Age, menopausal status, number of pregnancies, history of sex hormone use, BMI, physical activity, and education
Khan et al. 2004	Hokkaido	Japan	> 40	M: 1524 F: 1634	14·8	155	Cancer mortality	FFQ	C2 *v*. C1	RR: 0·80	0·50, 1·30	Age and smoking
						89				RR: 0·70	0·40, 1·30	Age, health status, health education, health screening and smoking
Tokui et al. 2005	JACC	Japan	40–79	M: 45 181 F: 62 643	11	344	Stomach cancer mortality	FFQ	C5 *v*. C1	RR: 0·82	0·50, 1·37	Age
						183				RR: 0·88	0·47, 1·64	
Nakanishi et al. 2021	Yamagata	Japan	40–74	F/M: 14 264	9	265	All-cause mortality	FFQ	C4 *v*. C1	HR: 0·62	0·42, 0·91	Age, gender, hypertension, diabetes mellitus, smoking, alcohol consumption, BMI and education period
					7	40	CVD mortality			HR: 1·06	0·39, 2·84	
					7	90	Cancer mortality			HR: 0·53	0·27, 0·99	
Lu et al. 2022	Miyagi	Japan	40–64	M: 16 565	25	4304	All-cause mortality	FFQ	> 3 times/week *v*. almost never	HR: 1·04	0·92, 1·17	Age, education level, BMI, smoking status, alcohol drinking status, history of hypertension, and history of diabetes, energy intake, fish intake, vegetable and fruit intake
						1048	CVD mortality			HR: 0·99	0·78, 1·26	
						1713	Cancer mortality			HR: 1·03	0·85, 1·24	
				F: 17 596		2522	All-cause mortality			HR: 0·92	0·81, 1·03	
						645	CVD mortality			HR: 0·87	0·69, 1·11	
						839	Cancer mortality			HR: 1·10	0·89, 1·34	
Lin et al. 2022	NHANES	USA	> 18	F/M: 32 625	8·1	3881	All-cause mortality	Food recall	Consumer *v*. non-consumer	HR: 0·83	0·71, 0·98	Age, sex, race, BMI, leucocytes count, Hb, platelet count, total bilirubin, creatinine, blood urea nitrogen, hypertension, diabetes, asthma, congestive heart failure, CHD, stroke, chronic bronchitis and cancer.
						651	CVD mortality			HR: 0·68	0·43, 1·08	
						863	Cancer mortality			HR: 1·00	0·72, 1·38	

RR, relative risk; F, female; g/d, gram(s) per d; HR, hazard ratio; serv, serving; NSCS, Nambour Skin Cancer Study; HPFS, Health Professionals Follow-Up Study; NHS, Nurses’ Health Study; GCS, Golestan Cohort Study; PURE, Prospective Urban Rural Epidemiology; EPIC-NL, European Prospective Investigation into Cancer and Nutrition – the Netherlands; RS, Rotterdam Study; NIH-AARP, National Institutes of Health-American Association of Retired Persons; JACC, Japan Collaborative Cohort Study; JMS, Jichi Medical School; NLCS, Netherlands Cohort Study; NHANES, National Health and Nutrition Examination Survey.

#### Findings from the systematic review

Out of twelve cohorts (eleven publications) investigating the association between yogurt consumption and overall mortality, four studies reported an inverse association^(2,12,27,28)^ and six showed no significant association^(16,25,26,47,49,50)^. However, in the publication of Schmid et al^(13)^, an inverse association was seen in women (NHS) but not men (HPFS). Moreover, in the study of Goldbohm et al.^(16)^, yogurt consumption was inversely associated with all-cause mortality among men but not women. In terms of CVD mortality, yogurt consumption was not associated with a lower risk of CVD mortality in nine cohorts^(13,14,25–28,47,49)^. However, two studies^(12,16)^ revealed that yogurt consumption was inversely related to deaths from CVD. For the association of yogurt consumption and cancer mortality, one publication^(28)^ reported an inverse association and nine did not find any significant association^(12,18–22,25,41,48,49)^. However, Schmid et al^(13)^ found an inverse association between yogurt intake and risk of cancer mortality among women (NHS) but not men (HPFS).

#### Findings from the meta-analysis

**Fig. 2:**
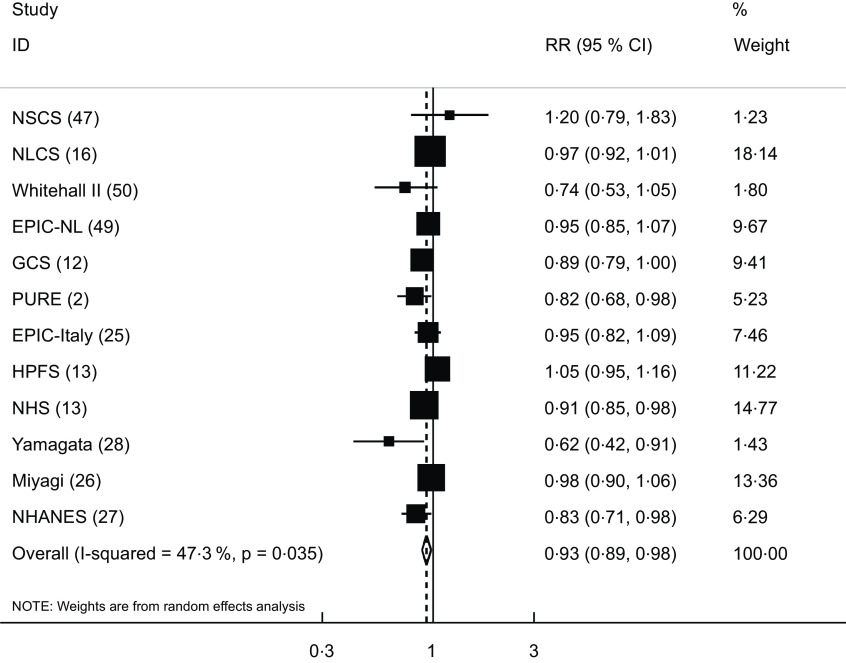
Forest plot for the association between yogurt consumption and risk of all-cause mortality in adults aged ≥ 18 years by comparing the highest and lowest categories of yogurt intake. RR, relative risk; NSCS, Nambour Skin Cancer Study; HPFS, Health Professionals Follow-Up Study; NHS, Nurses’ Health Study; GCS, Golestan Cohort Study; PURE, Prospective Urban Rural Epidemiology; EPIC-NL, European Prospective Investigation into Cancer and Nutrition-Netherland; NLCS, Netherlands Cohort Study; NHANES, National Health and Nutrition Examination Survey

The association between yogurt intake and risk of overall mortality was investigated in twelve cohort studies (eleven publications)^(2,12,13,16,25–28,47,49,50)^, which included a total of 476 160 participants with 75 791 cases. Comparing the highest *v*. lowest intakes of yogurt, the pooled RR for all-cause mortality was 0·93 (95 % CI: 0·89, 0·98), indicating a significant inverse association between yogurt consumption and overall mortality. There was evidence of moderate between-study heterogeneity (I^2^ = 47·3 %; *P* = 0·03) (Fig. 2).

**Fig. 3:**
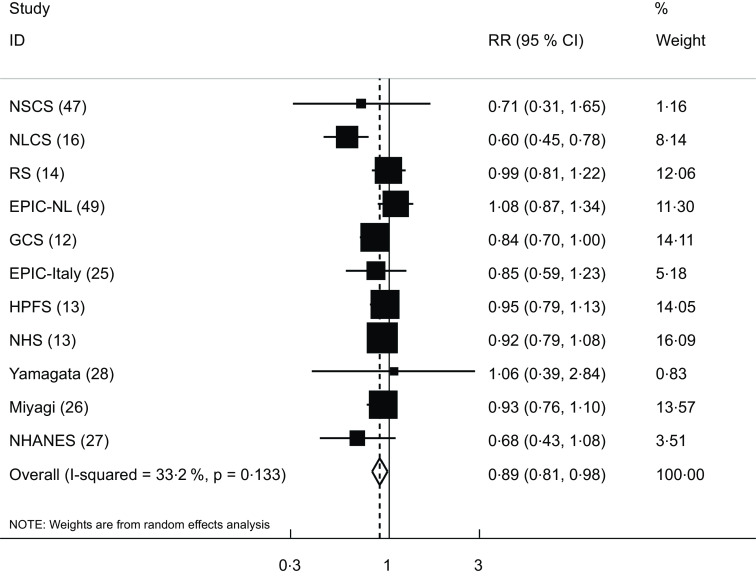
Forest plot for the association between yogurt consumption and risk of CVD mortality in adults aged ≥ 18 years by comparing the highest and lowest categories of yogurt intake. RR, relative risk; NSCS, Nambour Skin Cancer Study; HPFS, Health Professionals Follow-Up Study; NHS, Nurses’ Health Study; GCS, Golestan Cohort Study; EPIC-NL, European Prospective Investigation into Cancer and Nutrition-Netherland; RS, Rotterdam Study; NLCS, Netherlands Cohort Study; NHANES, National Health and Nutrition Examination Survey

Eleven cohort studies (ten publications)^(12–14,16,25–28,41,47,49)^ were included in the analysis of yogurt consumption and CVD mortality. These studies involved a total of 331 261 participants, among them 14 623 mortality cases were found. The pooled RR for CVD mortality was 0·89 (95 % CI: 0·81, 0·98), indicating a significant inverse association, with a low between-study heterogeneity (I^2^ = 33·2 %; *P* = 0·13) (Fig. 3).

**Fig. 4:**
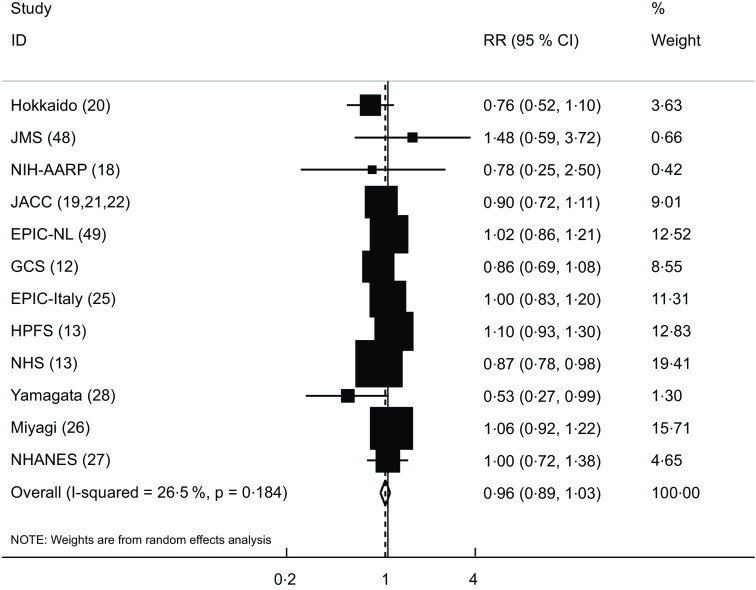
Forest plot for the association between yogurt consumption and risk of cancer mortality in adults aged ≥ 18 years by comparing the highest and lowest categories of yogurt intake. RR, relative risk; HPFS, Health Professionals Follow-Up Study; NHS, Nurses’ Health Study; GCS, Golestan Cohort Study; EPIC-NL, European Prospective Investigation into Cancer and Nutrition-Netherland; NIH-AARP, National Institutes of Health-American Association of Retired Persons; JACC, Japan Collaborative Cohort Study; JMS, Jichi Medical School; NHANES, National Health and Nutrition Examination Survey

The association between yogurt consumption and risk of cancer mortality was assessed in twelve studies (thirteen publications)^(12,13,18–22,25–28,48,49)^. These studies included a total of 741 973 participants with 20 926 deaths. Combining the RR from these publications, we failed to find any significant association between yogurt consumption and risk of cancer mortality (pooled RR: 0·96; 95 % CI: 0·89, 1·03). No significant between-study heterogeneity was also observed (I^2^ = 26·5 %; *P* = 0·18) (Fig. 4).

### Findings from the dose–response analysis

**Fig. 5:**
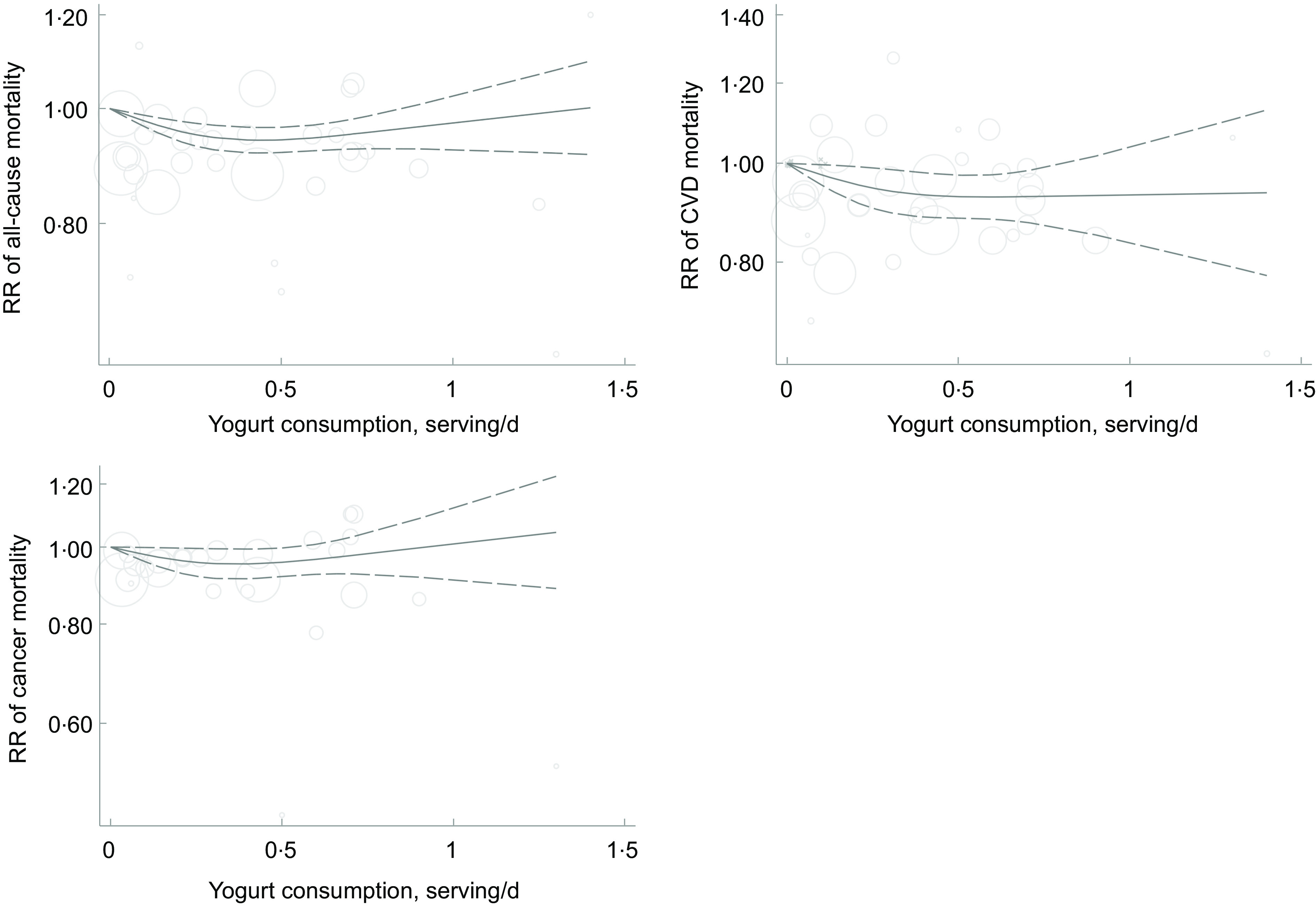
Non-linear dose–response association of yogurt consumption (based on serving/d) with risk of all-cause, CVD and cancer mortality in adults aged ≥ 18 years. The solid lines indicate the spline model. The dashed lines present the 95 % CI

Out of twelve studies investigating the association between yogurt consumption and overall mortality, eleven studies that provided sufficient information^(2,12,13,16,25,26,28,47,49,50)^ were included in the dose–response analysis. Each additional serving/d yogurt consumption was inversely associated with risk of all-cause mortality (pooled RR: 0·93; 95 % CI: 0·86, 0·99, I^2^ = 63·3 %) (online Supplementary Fig. 1). There was evidence of non-linear association between yogurt consumption and all-cause mortality (*P*-non-linearity < 0·001), and there was no further reduction in risk above 0·5 serving/d (Fig. 5).

Ten studies^(12–14,16,25,26,28,47,49)^ were included in the dose–response association of yogurt consumption and CVD mortality. Each additional serving/d yogurt consumption was associated with a 14 % lower risk of CVD mortality (pooled RR: 0·86; 95 % CI: 0·77, 0·97, I^2^ = 36·6 %) (online Supplementary Fig. 2). There was evidence of departure from linearity (*P*-non-linearity = 0·009), and there was no further reduction in risk above 0·5 serving/d (Fig. 5).

Out of twelve studies on the association between yogurt consumption and risk of cancer mortality, seven studies^(12,13,25,26,28,49)^ were included in the dose–response analysis. Each additional serving/d of yogurt consumption was not associated with cancer mortality (pooled RR: 0·95; 95 % CI: 0·85, 1·07, I^2^ = 53·3 %) (online Supplementary Fig. 3). There was no evidence of non-linear association (*P*-non-linearity = 0·08) (Fig. 5).

### Subgroup and sensitivity analyses, and publication bias

To examine the sources of between-study heterogeneity, we performed subgroup analysis. Table 2 shows findings for the different subgroups. None of the variables could explain the between-study heterogeneity, except for study location, which appeared to be the main factor responsible for this heterogeneity. The observed associations remained almost unchanged after control for important potential confounders, including BMI and energy intake. Based on sensitivity analysis, we found that the overall association did not affect by any individual study. Visual inspection of the funnel plot as well as findings from Begg’s and Egger’s tests revealed no evidence of publication bias in the analyses.

**Table 2:** Stratified analyses on associations of yogurt consumption with risk of mortality from all causes, CVD and cancer in adults aged ≥ 18 years

	n*	Pooled RR	95 % CI†	I^2^ (%)‡	*P*-heterogeneity§
All-cause mortality					
Location					
USA	3	0·93	0·83, 1·05	74·4	0·02
Non-USA	9	0·93	0·88, 0·99	38	0·11
Sex					
Male and female	8	0·88	0·82, 0·95	29·7	0·19
Male	3	1·00	0·92, 1·08	54·5	0·11
Female	3	0·94	0·89, 1·01	37·2	0·20
Follow-up duration					
> 10 years	9	0·96	0·92, 0·99	24·8	0·22
< 10 years	3	0·80	0·72, 0·90	0	0·38
Dietary assessment tools					
FFQ	11	0·94	0·90, 0·99	44·9	0·05
Food record	1	0·83	0·71, 0·98	–	–
Adjustment for energy					
Yes	10	0·95	0·91, 0·99	32·7	0·14
No	2	0·76	0·58, 0·99	46·3	0·17
Adjustment for BMI					
Yes	11	0·94	0·90, 0·99	45·5	0·04
No	1	0·82	0·68, 0·98	–	–
CVD mortality					
Location					
USA	3	0·91	0·82, 1·03	0	0·41
Non-USA	8	0·88	0·77, 1·01	46·5	0·07
Sex					
Male and female	7	0·92	0·83, 1·02	0	0·43
Male	3	0·87	0·67, 1·11	62·6	0·06
Female	3	0·83	0·68, 1·02	52·6	0·12
Follow-up duration					
> 10 years	9	0·90	0·82, 0·99	40·3	0·09
< 10 years	2	0·74	0·48, 1·12	0	0·42
Dietary assessment tools					
FFQ	10	0·90	0·82, 0·99	33·3	0·14
Food record	1	0·68	0·43, 1·08	–	–
Adjustment for energy					
Yes	9	0·90	0·82, 0·99	40·3	0·09
No	2	0·74	0·48, 1·12	0	0·42
Adjustment for BMI					
Yes	11	0·89	0·81, 0·98	33·2	0·13
No	–	–	–	–	–
Cancer mortality					
Location					
US	4	0·97	0·83, 1·13	44·3	0·14
Non-USA	8	0·96	0·87, 1·05	24·6	0·23
Sex					
Male and female	6	0·96	0·86, 1·08	12·4	0·33
Male	5	0·97	0·83, 1·14	29·9	0·22
Female	4	0·96	0·81, 1·13	46·6	0·13
Follow-up duration					
> 10 years	7	0·97	0·89, 1·06	42	0·11
< 10 years	5	0·90	0·76, 1·08	3·2	0·38
Dietary assessment tools					
FFQ	11	0·96	0·88, 1·04	32·9	0·13
Food record	1	1·00	0·72, 1·38	–	–
Adjustment for energy					
Yes	7	0·98	0·90, 1·06	32·2	0·18
No	5	0·88	0·73, 1·05	16·5	0·31
Adjustment for BMI					
Yes	9	0·97	0·89, 1·06	34·5	0·14
No	3	0·88	0·73, 1·06	0	0·39

RR, relative risk.

* Number of relative risks.

† Obtained from the random-effects model.

‡ Inconsistency – the percentage of variation across studies due to heterogeneity.

§ Obtained from Q test.

## Discussion

The findings from the current systematic review and meta-analysis of seventeen cohort studies demonstrated a significant inverse association between yogurt consumption and mortality from all causes and CVD. However, we failed to find any statistically significant association between yogurt consumption and cancer mortality. Each additional serving of yogurt consumption per d was associated with a lower risk of mortality from all causes and CVD.

Although the association between total dairy consumption and risk of mortality has been widely examined, less attention has been paid to the association between yogurt consumption and mortality. To the best of our knowledge, this is the most comprehensive meta-analysis of cohort studies that investigated the association of yogurt consumption with the risk of mortality. In the present study, we found a significant inverse association between yogurt consumption and risk of all-cause mortality. Our findings are comparable with a meta-analysis on the association between dairy intake and mortality. A meta-analysis of prospective studies, including 636 726 participants, reported a significant inverse association between consumption of fermented dairy products and risk of all-cause mortality^(41)^. In contrast, no association was observed between yogurt intake and overall mortality in a meta-analysis of prospective cohorts^(51)^. Similar results were also found in another meta-analysis on yogurt consumption and risk of all-cause mortality^(17)^. The different findings might be explained by some methodological limitations of that meta-analysis^(17)^. The authors included an ineligible study in which the RR was reported for the combination of yogurt and cottage cheese, not yogurt only^(23)^. They also included a prospective study conducted on cancer patients^(24)^. In addition, we included further studies^(13,25–28)^ that appeared after the release of those meta-analyses^(17,51)^.

In the current study, we found that yogurt consumption was significantly and inversely associated with the risk of CVD mortality. Unlike our findings, yogurt intake was not related to CVD mortality in the latest meta-analysis in this regard^(17)^. Lack of such an association was also observed in another meta-analysis of prospective cohort studies^(51)^. It should be noted that several large cohort studies^(13,25–28)^ have been published since the release of previous meta-analyses^(17,51)^. This might explain the diverse findings. With regard to cancer mortality, our findings were in agreement with a prior meta-analysis examining the association with yogurt consumption^(17)^. Lu et al^(52)^ in a meta-analysis of eleven population-based cohort studies reached no significant association between consumption of dairy products and risk of cancer mortality. It is possible that the association between yogurt consumption and cancer mortality is dependent on the cancer type and characteristics of the study population. Other potential factors including different lifestyle-related factors and dietary intakes might need to be further explored in future studies.

Although the underlying mechanisms behind the inverse association between yogurt consumption and mortality risk are not fully understood, one possible explanation is alteration in the gut microbiome. Yogurt carries the beneficial bacteria into the gut-promoting immune functions through which it can improve human health. Lisko et al.^(53)^ demonstrated a fluctuation in the diversity of gut microbiota after a short-term period of yogurt intake in healthy subjects, indicating that regular consumption of yogurt may favourably affect the gut microbiota. However, the positive effects of yogurt intake are not limited to bacterial quantity and diversity. In fact, the beneficial metabolites produced by bacteria such as SCFA may also play an important role in its beneficiary effect^(13,53)^.

Regular consumption of fermented yogurt products in experimental studies has been associated with an increased level of HDL-cholesterol^(54)^ as well as lower levels of total cholesterol and LDL-cholesterol. Therefore, a reduction in the ratio of LDL-cholesterol:HDL-cholesterol, an index of atherogenicity, might explain its inverse association with mortality^(55)^. Moreover, fermented dairy products serve as an important dietary source of vitamin K2 (menaquinone), which can in turn stimulate β-cell proliferation and improve insulin sensitivity^(56)^. Yogurt intake was longitudinally associated with less weight gain and lower waist circumference, supporting the growing evidence that changes in gut bacteria may affect weight gain^(57)^.

Health-promoting effects of fermented yogurt products may be attributed to the biosynthesis or release of bioactive peptides with antihypertensive, antimicrobial, antioxidative and immune-modulatory properties^(13,15)^. Findings from clinical trials have also indicated that consumption of yogurt may be effective in reducing chronic inflammation^(58,59)^. Ca content of yogurt might also play a role in its inverse association with mortality. Ca appears to interact with SFA, forming fatty acid-insoluble soaps, consequently reducing SFA absorption, lowering TAG concentrations and improving the HDL-cholesterol:LDL-cholesterol ratio. Diets rich in Ca were associated with beneficial changes in blood pressure and lowering risk of stroke^(60,61)^. Taken together, these findings support the notion that yogurt consumption may be effective in reducing risk of all-cause and CVD mortality.

The present meta-analysis has several strengths. A large sample size (896 871 participants and 75 791 cases) provides an adequate level of statistical power to detect the associations of interest. Moreover, findings were adjusted for numerous potential confounding variables in the included studies. Publication bias that can affect the results of studies is possible in any meta-analysis, but we found no evidence of such bias. Furthermore, a dose–response analysis in the current study adds to the present literature. However, a number of limitations should be considered. First, there was not sufficient information available to investigate the association of yogurt consumption with risk of mortality based on its fat content. Second, the present meta-analysis was based on observational studies; therefore, causality cannot be inferred. Third, given the use of FFQ as a method of dietary assessment in most included studies, measurement errors and misclassification of participants in terms of yogurt intake cannot be ignored. Fourth, the units of yogurt consumption were different across studies. Fifth, most included studies had measured yogurt consumption only at study baseline. Sixth, some studies did not provide sufficient information for the dose-dependent meta-analysis. Moreover, nutrient content of yogurt is dependent on various factors such as animals’ diet, food fortification, biosynthesis and physicochemical conditions, which might be different across studies.

In conclusion, we found an inverse association between yogurt consumption and risk of all-cause and CVD mortality; however, we failed to find any significant association with cancer mortality. To shed light on this issue, it seems that further studies, particularly pooled analyses, are required.

## Supplementary material

For supplementary material accompanying this paper visit https://doi.org/10.1017/S1368980022002385. click here to view supplementary material
