# Supplementary material for: Yogurt consumption and risk of mortality from all causes, CVD and cancer: a comprehensive systematic review and dose–response meta-analysis of cohort studies
Source: Public Health Nutr. 2022 Nov 9;26(6):1196–209. doi: 10.1017/S1368980022002385 (PMC10346031; doi:10.1017/S1368980022002385)
Supplement: Supplementary file 1 [file S1368980022002385sup001.docx]

**Online Supporting Material**

**Supplemental Table 1**: Quality assessment of cohort studies included in the current systematic review and meta-analysis on the association between yogurt consumption and mortality based on the Newcastle-Ottawa scale

| Author | Representativeness of the exposed cohort | Selection of the non-exposed cohort | Ascertainment of exposure | Outcome of interest was not present at the start of the study | Energy adjustment | Controls for any additional factor | Assessment of outcome | Follow-up long enough | Adequacy of follow-up of cohorts | Total |
| --- | --- | --- | --- | --- | --- | --- | --- | --- | --- | --- |
| Soedamah-Muthu et al.2013 | * | * |  | * | * | * | * | * | * | 8 |
| Bonthuis et al. 2010 |  | * |  | * | * | * | * | * | * | 7 |
| Schmid et al. 2020 | * | * |  | * | * | * | * | * | * | 8 |
| Farvid et al.2017 | * | * | * | * |  | * | * | * | * | 8 |
| Dehghan et al.2018 | * | * |  | * | * | * | * |  | * | 7 |
| Praagman et al.2015 | * | * |  | * | * | * | * | * | * | 8 |
| Pala et al.2019 | * | * |  | * | * | * | * | * | * | 8 |
| Praagman et al.2014 | * | * |  | * | * | * | * | * | * | 8 |
| Park et al.2007 | * | * |  | * | * | * | * |  | * | 7 |
| Kojima et al. 2014 | * | * |  | * |  | * | * |  | * | 6 |
| Matsumoto et al. 2007 | * | * |  | * |  | * | * |  | * | 6 |
| Goldbohm et al. 2011 | * | * |  | * | * | * | * | * | * | 8 |
| Sakauchi et al. 2007 | * | * |  | * |  | * | * | * | * | 7 |
| Khan et al. 2004 | * | * |  | * |  | * | * | * | * | 7 |
| Tokui et al. 2005 | * | * |  | * |  |  | * | * | * | 6 |
| Nakanishi et al. 2021 | * | * |  | * |  | * | * |  | * | 6 |
| Lu et al. 2022 | * | * |  | * | * | * | * | * | * | 8 |
| Lin et al. 2022 | * | * |  | * |  | * | * |  | * | 6 |

**Online Supporting Material**

**Supplemental Figure 1**





Forest plot for the risk of all-cause mortality based on one serving/day increase in yogurt consumption in adults aged >18 years. Horizontal lines represent 95% CIs. Diamonds represent the pooled estimates from the random-effects analysis. RR: relative risk, CI: confidence interval, NSCS: Nambour skin cancer study; HPFS: Health Professionals Follow-Up Study; NHS: Nurses' Health Study; GCS: Golestan Cohort Study; PURE: Prospective Urban Rural Epidemiology; EPIC- NL:European Prospective Investigation into Cancer and Nutrition –NLCS: Netherlands Cohort Study.

**Online Supporting Material**

**Supplemental Figure 2**





Forest plot for the risk of CVD mortality based on one serving/day increase in yogurt consumption in adults aged >18 years. Horizontal lines represent 95% CIs. Diamonds represent the pooled estimates from the random-effects analysis. RR: relative risk, CI: confidence interval, NSCS: Nambour skin cancer study; HPFS: Health Professionals Follow-Up Study; NHS: Nurses' Health Study; GCS: Golestan Cohort Study; EPIC-NL: European Prospective Investigation into Cancer and Nutrition-Netherland; RS: Rotterdam Study; NLCS: Netherlands Cohort Study.

**Online Supporting Material**

**Supplemental Figure 3**





Forest plot for the risk of cancer mortality based on one serving/day increase in yogurt consumption in adults aged >18 years. Horizontal lines represent 95% CIs. Diamonds represent the pooled estimates from the random-effects analysis. RR: relative risk, CI: confidence interval, HPFS: Health Professionals Follow-Up Study; NHS: Nurses' Health Study; GCS: Golestan Cohort Study; EPIC- NL: European Prospective Investigation into Cancer and Nutrition-Netherland.
